# Urine from Treated Cattle Drives Selection for Cephalosporin Resistant *Escherichia coli* in Soil

**DOI:** 10.1371/journal.pone.0048919

**Published:** 2012-11-07

**Authors:** Murugan Subbiah, Devendra H. Shah, Thomas E. Besser, Jeffrey L. Ullman, Douglas R. Call

**Affiliations:** 1 Department of Veterinary Microbiology and Pathology, Washington State University, Pullman, Washington, United States of America; 2 Paul G. Allen School for Global Animal Health, Washington State University, Pullman, Washington, United States of America; 3 Department of Agricultural and Biological Engineering, University of Florida, Gainesville, Florida, United States of America; Cornell University, United States of America

## Abstract

The U.S. Food and Drug Administration recently issued new rules for using ceftiofur in food animals in part because of an increasing prevalence of enteric bacteria that are resistant to 3^rd^-generation cephalosporins. Parenteral ceftiofur treatment, however, has limited effects on enteric bacteria so we tested the hypothesis that excreted ceftiofur metabolites exert significant selection pressure for ceftiofur-resistant *Escherichia coli* in soil. Test matrices were prepared by mixing soil with bovine feces and adding urine containing ceftiofur metabolites (CFM) (0 ppm, ∼50 ppm and ∼100 ppm). Matrices were incubated at 23°C or 4°C for variable periods of time after which residual CFM was quantified using a bioassay. *Bla*_CMY-2_ plasmid-bearing ceftiofur resistant (cef^R^) *E. coli* and one-month old calves were used to study the selection effects of CFM and transmission of cef^R^ bacteria from the environment back to animals. Our studies showed that urinary CFM (∼13 ppm final concentration) is biologically degraded in soil within 2.7 days at 23°C, but persists up to 23.3 days at 4°C. Even short-term persistence in soil provides a >1 log_10_ advantage to resistant *E. coli* populations, resulting in significantly prolonged persistence of these bacteria in the soil (∼two months). We further show that resistant strains readily colonize calves by contact with contaminated bedding and without antibiotic selection pressure. Ceftiofur metabolites in urine amplify resistant *E. coli* populations and, if applicable to field conditions, this effect is far more compelling than reported selection *in vivo* after parenteral administration of ceftiofur. Because ceftiofur degradation is temperature dependent, these compounds may accumulate during colder months and this could further enhance selection as seasonal temperatures increase. If cost-effective engineered solutions can be developed to limit *ex vivo* selection, this may limit proliferation for ceftiofur resistant enteric bacteria while preserving the ability to use this important antibiotic in food animal production.

## Introduction

Antibiotic resistance is a significant public health concern and in response the U.S. Food and Drug Administration has issued new rules for cephalosporin use in food animals [1]. In the U.S. resistance to 3^rd^-generation cephalosporins is most often mediated by a *bla*_CMY-2_ gene that is harbored by IncA/C plasmids in enteric bacteria [2], [3]. The prevalence of *bla*_CMY-2_ plasmid-bearing *Salmonella* and *Escherichia coli* has increased rapidly in the U.S. cattle population over the last decade [4], [5], [6], [7], [8], [9]. For instance, Daniels et al. [10] found that at least 97% of dairy herds in Washington State were positive for *bla*_CMY-2_ plasmid-bearing *E. coli* and the U.S. Department of Agriculture [11] reported that the percentage of *Salmonella* sp. with ceftiofur resistance isolated from cattle increased from 0% in 1997 to 21.6% in 2005. In addition, studies conducted by Subbiah et al. [12] showed that long-term maintenance of *bla*_CMY-2_ plasmids in resistant *E. coli* hosts require some level of selection pressure, and ceftiofur use in livestock has been implicated in this process [13], [14].

Ceftiofur is an injectable 3^rd^-generation cephalosporin that is used to treat respiratory infections, metritis, and pododermatitis in cattle. The use of ceftiofur in food animals has increased due to its high effectiveness, convenient formulations, short withholding, and increased label indications. This increased use has been accompanied by a parallel increase in the prevalence of ceftiofur resistant (cef^R^) enteric bacteria in food animal populations [13], [14], [15], [16], [17].

Following injection ceftiofur is converted mostly into desfuroylceftiofur, a pharmacologically active metabolite of ceftiofur (CFM) [15]. Within 24 h the majority of the CFM is excreted into feces (∼30%) and urine (∼70%) [18]. CFM is degraded in cattle feces [19] and this may explain why *in vivo* selection of resistant enteric bacteria is limited and inconsistent in cattle [8], [10], [13], [14], [17]. Subbiah et al. [20] reported that several β-lactams, including cephalosporins, remain bactericidal in soils >24 h suggesting the possibility that primary selection for cef^R^ *E. coli* could occur *ex vivo* rather than *in vivo*; if this hypothesis is correct it could indicate new opportunities to mitigate the proliferation of cef^R^ bacteria. To test this hypothesis we needed to first determine how long and under what conditions CFM remain bioavailable in the environment, and determine if CFM can influence the soil microbiota by proportionally or numerically enriching resistant bacterial populations. Finally, for this model to be feasible it must be possible for resistant bacteria to colonize food animals by contact with the soil stratum.

In this study we examined the fate of CFM in soil and found that temperature is the most important mitigating variable with an average 50 parts per million (ppm) CFM in urine (∼13 ppm in manure-soil matrix) remaining biologically available from days to weeks depending on conditions tested. Temperature primarily affects the rate of loss where the soil microflora appears to be responsible for degrading CFM. The fact that CFM is biodegraded means that there may be ways to engineer novel mitigation solutions. Importantly, however, even a short duration of bioavailable CFM is sufficient to provide a numerical advantage to cef^R^ *E. coli*; an advantage that remains evident for months thereby increasing the likelihood of transmission back to food animals. This is the first study demonstrating that *ex vivo* selection by excreted CFM could very likely play a role in the emergence, perpetuation and dissemination of ceftiofur resistance in food animal populations.

## Materials and Methods

### Ethics Statement

All animal studies described in this report were approved by the Washington State University Institutional Animal Care and Use Committee (Animal Subjects Protocol #04013-001).

### Primary Reagents

Two bacterial strains, nalidixic acid resistant (nal^R^) *bla*_CMY-2_ positive *E. coli* H4H (multidrug resistant, including to ceftiofur with a minimum inhibitory concentration [MIC] = 20 ppm) [12] and *E. coli* K-12, were used in this study. Two type of soils were used, which included a low pH (4.7), high organic content (2.27%) silt-loam and a high pH (7.75), low organic content (0.24%) sand [20]. Key reagents included McConkey agar, Luria-Bertani broth and buffered peptone powder (Becton, Dickinson and Co, Sparks, MD), dimethyl sulfoxide (DMSO) and calcium chloride (JT Bakers, Phillipsburg, NJ), nalidixic acid (MP Biomedicals, LLC, Illkrich, France), ceftiofur (Sigma-Aldrich, St. Louis, MO), desfuroylceftiofur (Toranto research chemicals, Ontario, Canada), ceftiofur sodium (Naxcel, Pharmacia & Upjohn Co., NewYork, NY).

### Matrix Components

To determine how long CFM remains bioavailable in soil, we mixed soil (one of two types), feces and urine (with 0, 50 and 100 ppm CFM) and measured the bioactivity of CFM over time. Urine used in these experiments was collected from a single month-old male calf and was collected aseptically in sterile bottles both before and after administration of a single dose of ceftiofur sodium (Naxcel, 2.2 mg/kg body weight, intramuscular). El Gendy et al. reported that after intramuscular administration of ceftiofur calves excreted most of the ceftiofur metabolites in the first 4 h post-injection and thus we collected urine up to 8 h post-injection [21]. The collected urine was filter-sterilized (0.22 µm pore size) and stored at –20°C until further use.

The approximate concentration (ppm; equivalent to µg/ml if in liquid phase) of CFM in urine was estimated by comparing two-fold dilution curves of urine from the ceftiofur treated calf against two-fold dilution curves of urine that had been spiked with either ceftiofur or desfuroylceftiofur. The stock solutions of ceftiofur (20 mg/ml) and desfuroylceftiofur (5 mg/ml) were prepared in DMSO and stored at −20°C. We mixed 100 µl of each urine dilution and 100 µl of 2X Luria-Bertani broth containing *E. coli* K-12 strain (10^6^/ml; minimum inhibitory concentration for CFM = 0.5 ppm) in 100-well plates (3 wells per sample). The loaded plates were covered and incubated for 24 h at 37°C in an optical density (OD) plate reader and the measurements (OD_595_) were collected at regular intervals (2 h) [20]. The urine samples that inhibited *E. coli* equivalent to approximately 50 ppm and 100 ppm of the desfuroylceftiofur and ceftiofur standards were chosen for further studies (both standards produced similar dose-response curves).

Fresh fecal samples were collected in sterile conical tubes from dairy cattle from the Washington State University Dairy. Individual fecal samples vary in their inherent ability to inhibit *E. coli* growth (unpublished observation). To avoid confounding variables, fecal samples were first tested for inhibitory action upon *E. coli* K-12 by mixing feces from individual animals with soil (1∶25, feces:soil) and saturating with urine that was free of excreted antibiotics. The matrices were then incubated at room temperature (23°C) for 1 h. An equal amount of each matrix (0.5 g) and nanopure water (500 µl) were mixed (vortexed for 15 sec and then rocked for 1 h at 300 rpm on a shaker) in a 15 ml conical tube. After mixing, the tubes containing slurries were centrifuged at 4,000 rpm for 20 min and the supernatants were decanted and filter-sterilized (0.45 µm) into a 1.5 ml tube and tested for inhibitory activity using *E. coli* K-12 as described above (12 h incubation instead of 24 h). The non-inhibitory fecal samples (OD_595_∼1.0) were pooled together and used for further studies.

### Matrix Preparation

Matrices were prepared by adding 24 g soil and 1 g fresh feces (1∶25, feces to soil) in glass petri plates, after which one set (n = 12) of plates was autoclaved (dry cycle for 15 min, 121°C at 15 psi) and the other set of plates was not autoclaved. Urine containing 0 ppm (collected from an untreated calf; see above), 50 ppm or 100 ppm CFM (collected from the same calf after receiving a ceftiofur injection) was added into each petri plate containing soil and feces until the soil was saturated but not to the point of being a liquid slurry (∼6–6.5 ml). The final concentrations of CFM in this experimental system were 0, ∼13 and ∼26 ppm (µg/g matrix). Each matrix was mixed thoroughly and incubated in humidified chambers (>65% humidity) covered with aluminum foil to maintain a dark environment. Samples were stored at 23°C (21.5°C–23°C) and at 4°C (3.8°C–4.1°C). A total of 24 experimental treatments were considered, based on combinations of 2 soil types, 3 CFM concentrations, autoclaved and non-autoclaved soil-manure matrices, and 2 temperatures, with each treatment including three independent replicates. To determine the role of feces and soil in the deactivation of CFM, matrices were prepared using different combinations of soil (two types, autoclaved and non-autoclaved), feces (none, autoclaved and non-autoclaved), urine (0, 50 and 100 ppm) and temperature (4°C and 23°C).

### Assessing CFM Bioactivity from Matrices

CFM bioactivity in the soil-manure matrices was assessed using the protocol from Subbiah et al. [20] with slight modifications. Briefly, subsamples of each matrix were collected (∼0.5 to 1.5 g) in a 15 ml conical tube every day for the first week of the experiment and then less frequently until the antimicrobial activity of CFM was lost or the matrices became desiccated. An equal amount of 0.01 M calcium chloride amended nanopure water was added into the subsamples and mixed well by vortexing for 15 sec. The tubes were wrapped in aluminum foil and rocked in a shaker (45° angle slanting position) for ∼2 h at room temperature. After shaking, the slurries were centrifuged at 4,000 rpm for 20 min and the supernatants were decanted, filter-sterilized (0.45 µm pore size) and stored (−20°C) or tested immediately using an *E. coli* K-12 bioassay.

### *E. coli* K-12 Bioassay

Filter-sterilized supernatants were added 1∶1 into 2X LB media with *E. coli* K-12 (10^6^ cfu/ml) that was contained in 100-well plates (5 wells per sample; 200 µl total volume per well). Plates were covered and incubated in an optical density (OD) plate reader (Boioscreen, Torrance, CA) at 37°C for 24 h with OD_595_ readings collected every hour. The day at which filter-sterilized supernatant no longer constrained *E. coli* K-12 growth to <0.5 OD_595_ was considered the day when inhibition by CFM was no longer biologically relevant; this day was designated as D_50._

### Assessing the Effect of Hydrolysis on the Deactivation of Ceftiofur Metabolites

To determine the effect of hydrolysis and the role of urine components on deactivation of CFM, we aliquoted 15 ml of urine with 0, 50 and 100 ppm CFM in tubes. The tubes were covered with aluminum foil and incubated at 23°C and 4°C for the duration of our experiments. These samples were then processed in parallel with the matrix samples.

### Determine if Ceftiofur Metabolites in Soil Confer a Selective Advantage to cef^R^ *E. coli*

The colony forming unit (cfu) were enumerated to study the survival pattern of nalidixic acid resistant (nal^R^) cef^R^ *E. coli* (strain H4H) and fecal coliforms (mostly *E. coli*) in soil matrices under various conditions [12]. The matrices (autoclaved and non-autoclaved) were prepared as described above, except only the sandy soil was used for these experiments. Feces (1g) were added to the soil, one set was autoclaved and another was not autoclaved. Urine (with 0 and 50 ppm CFM) was added until the soil was saturated (∼6.5 ml) and mixed well in glass petri plates (final concentration of CFM ∼13 ppm). *E. coli* strain H4H (nal^R^, cef^R^) was cultured overnight in LB broth and was then added (∼100 µl) to the matrix and mixed well. The final H4H concentration was between 10^5^ and 10^7^ cells per g matrix on day 0. The matrices were incubated in aluminum foil covered humid chambers at room temperature and at 4°C for several weeks.

Subsamples from each treatment were collected every day for the first three days and less frequently thereafter. The cfu of H4H was calculated for the subsamples using the method described by Subbiah et al. [12]. Briefly, slurries were prepared separately for each matrix by mixing 0.5 g of subsample matrix with 4.5 ml of sterile buffered peptone water (1∶10 dilution, detection limit ≥1,000 cells per g matrix) in 15 ml conical tubes and then serially diluted (10-fold) in sterile buffered peptone water. The diluted slurry was plated on McConkey agar plates containing nalidixic acid (20 ppm) and ceftiofur (10 ppm) and incubated overnight at 37°C. After overnight incubation the cfu of H4H *E. coil* was enumerated per g of matrix. Cell counts were determined for 140 days and regression analysis was used to estimate the date, on average, when the cfu would drop below 10 cells per g matrix (see below).

We also determined if CFM affects the native ceftiofur susceptible (cef^S^) fecal coliform (predominantly *E. coli* based on colony morphology) population at room temperature. To determine the survival of native cef^R^ and cef^S^ fecal *E. coli* we used non-autoclaved matrices without adding any additional bacteria. Matrices were screened for cef^R^ *E. coli* at the outset of the experiments and matrices were only used if they were negative for existing cef^R^ *E. coli*. The cfu of both cef^R^ and cef^S^ fecal *E. coli* was calculated by using McConkey agar plates with and without ceftiofur (5 µg/ml) on days 0, 1, 3, 7, 14 and 21. During the incubation period if the cfu dropped below 1,000 the dilution was changed from 1∶10 to 1∶1 dilution to achieve a detection limit of ≥10 cells per g matrix.

### Transmission of bla_CMY-2_ Plasmid-bearing *E. coli* from the Environment to Calves

To determine if cattle can acquire bacteria from topsoil we conducted an animal experiment in two rooms bedded with fresh compost. In one room, *bla*_CMY-2_ positive *E. coli* strain AR060302 was sprayed on the bedding (10^4^ cells per ml LB broth) using a 1 gallon garden sprayer and the other room was left unsprayed. After spraying (30 min), two one-month old Holstein-Friesian calves that were culture negative for cef^R^ and florfenicol resistant (flo^R^) *E. coli* were housed in each room and fed *ad libitum* with alfalfa hay, calf pellets and water. Fresh rectal fecal samples and bedding materials (top layer) were collected aseptically in sterile conical tubes on days 0, 2, 4, and 7. The cfu of flo^R^cef^R^ AR060302 *E. coli* was calculated as described above (McConkey agar plates with florfenicol 40 µg/ml and ceftiofur 10 µg/ml) and the strain identity was confirmed by antibiotic resistant pattern and presence of a plasmid of the expected size using methods described elsewhere [12].

### Statistical Analysis

Triplicate D_50_ (days of CFM bioactivity) values were compared for all the conditions tested. Fixed effects ANOVA was used to compare the main effects (soil type, CFM concentration, feces, autoclaving and temperature). Bacterial counts were log-transformed to meet assumptions of normality and when assumptions of normality could be met, ANOVA was used with a Tukey-Kramer multiple-comparison test. When normality was not met (based on modified-Levene equal variance test, α = 0.05), a Kruskal-Wallis non-parametric test was used to compare results. ANOVA calculations were made using NCSS 2007 (NCSS, LLC. Kaysville, UT). Linear regression was used to model the decline in *E. coli* populations during long-term experiments. Three independent replicate measurements were collected for each time point. Averaged replicates (n = 8 discreet time points) were analyzed using SigmaPlot ver. 12.2 (Systat Software, Inc., San Jose, CA) and subject to a Shipiro-Wilk test (normality of residuals) and a constant variance test. The slopes the two regression models were compared using the parallel line analysis function provided by the SigmaPlot software. Extrapolations of the regression model using a 95% lower confidence interval were calculated using NCSS 2007 to estimate time of population extinction.

## Results

We prepared matrices composed of soil (sandy or silt loam [20]), cattle urine with 0 ppm, 50 ppm, 100 ppm CFM, and cattle feces (1∶25 feces:soil). To quantify CFM residues, matrices were mixed with water (50∶50 w/v) and filter-sterilized supernatant from these slurries was added to ceftiofur susceptible *E. coli* K-12 culture [20]. Growth of the bacteria was quantified by measuring optical density (OD) over 24 h. During time-course experiments the day at which the *E. coli* K-12 density exceeded ≥0.5 OD_595_ was considered the day when inhibition was negated (designated as D_50_).

### Ceftiofur Metabolites Remain Bioactive in Soil Matrices

Using this experimental design we found no significant difference between D_50_ for the silt-loam and sandy soil types (*P* = 0.93), and there was an expected dose response with D_50_ being greater for 100 ppm compared to 50 ppm (*P* = 0.044). Subsequent analyses used sandy soil and 50 ppm CFM in urine as a standard concentration (CFM concentrations in urine range from ∼7 to 165 ppm within 24 h of ceftiofur administration [21]). The D_50_ value of filter-sterilized urine alone with 50 ppm CFM was >110 days at both 23°C and 4°C indicating that abiotic hydrolysis of CFM was negligible during the course of these experiments.

### Ceftiofur Metabolite Bioactivity is Extended at Lower Temperature with Autoclaved Soil

**Figure 1:**
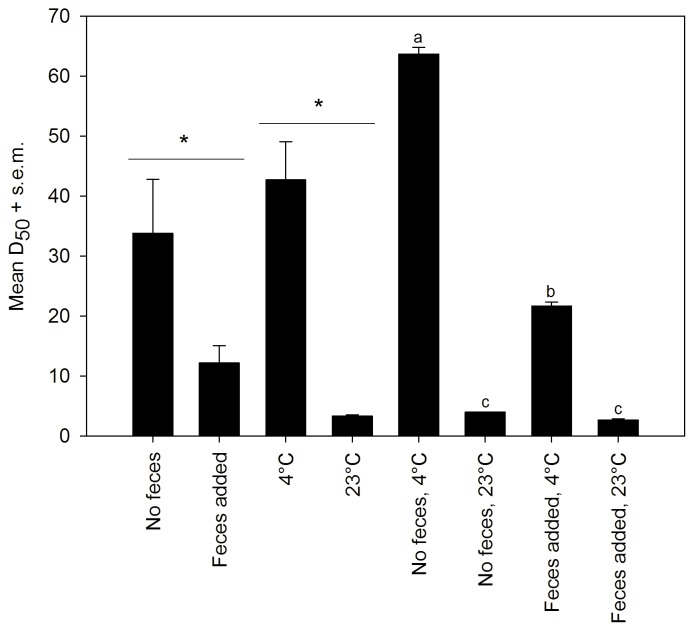
Ceftiofur metabolites remain biologically available in topsoil under different conditions. Effect of temperature and addition of cattle feces (1∶25) on ceftiofur metabolites (CFM) as measured by recovery of growth for *E. coli* K-12 (D_50_). Main effects and interactions were significant (*P*<0.0001; two-factor ANOVA). Letters designate group differences based on Tukey-Kramer multiple comparison test (*P*<0.001). Bars = s.e.m.

Temperature had the largest effect on D_50_ with a ∼13-fold increase from 23°C to 4°C (*P*<0.0001; Fig. 1). There was a significant interaction between temperature and feces (*P*<0.0001) where addition of feces at 23°C had no effect on D_50_ (P>0.001), but at 4°C there was a ∼3-fold reduction in D_50_ (*P*<0.001; Fig. 1). The temperature dependence of D_50_ suggests that CFM is lost through biological degradation where addition of feces accelerates this effect at lower temperatures.

### Soil Microflora is Responsible for Loss of CFM

**Figure 2:**
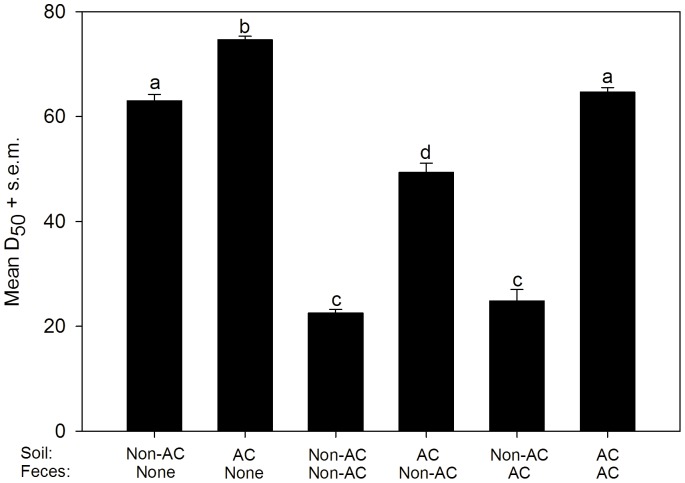
Soil mircoflora is responsible for degradation of ceftiofur metabolites. Effect of autoclaved (AC) vs. non-autoclaved (non-AC) soil and feces on ceftiofur metabolites (CFM) as measured by recovery of growth for *E. coli* K-12 (D_50_). Sandy soil was used and chambers were incubated at 4°C. Main effects (soil and feces) were significant (*P*<0.0001). Letters designate group differences based on Tukey-Kramer multiple comparison test (*P*<0.001). Bars = s.e.m.

Autoclaving the matrix clearly extended the duration of CFM bioactivity. To determine how feces contributed to this process we incubated urine with 50 ppm CFM at 4°C in soil (autoclaved or non-autoclaved), and with or without autoclaved or non-autoclaved feces (Fig. 2). Autoclaving soil resulted in significantly higher D_50_ values (*P*<0.001), but addition of feces (autoclaved or not) caused an equivalent reduction in D_50_. Addition of non-autoclaved feces to autoclaved soil showed that fecal microflora may contribute to biodegradation of ceftiofur (Fig. 2), although this could be a nutrient effect because the autoclaved soils we used were not completely sterilized. Consequently, it appears that CFM in the soil matrix is degraded primarily by the soil microflora and to a lesser extent by fecal microflora.

### Ceftiofur Metabolites in Urine Select for Ceftiofur Resistant *E. coli* in Soil

**Figure 3:**
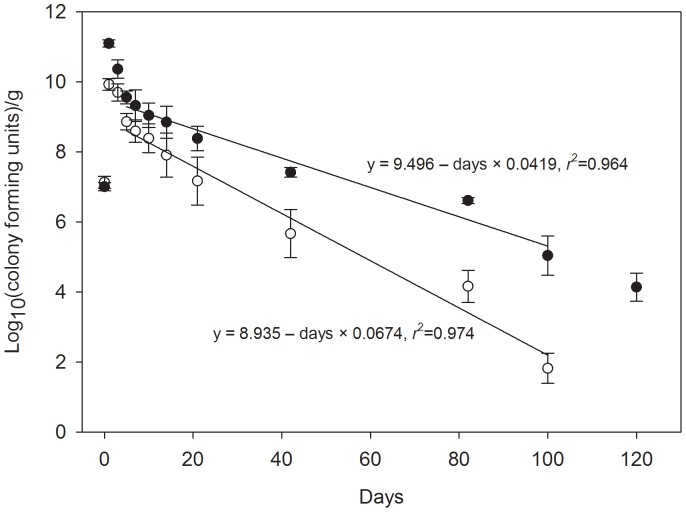
Ceftiofur metabolites select for ceftiofur resistant *E. coli* in soil. Mean colony forming units for cef^R^ *E. coli* after incubation at 23°C in a sandy soil and urine matrix (open circles, 0 ppm CFM; closed circles 50 ppm CFM). Regression lines between days 5 and 100 were significantly different (*P*<0.008). Bars = s.e.m.

To determine if CFM confers a selective advantage to cef^R^ *E. coli*, we studied the survival of a *bla*_CMY2_ plasmid-bearing *E. coli* (strain H4H) at 23°C in soil with urine containing 0 or 50 ppm CFM (Fig. 3). Colony counts (cfu) increased 2.8 log or 4.1 log, respectively, within 24 h of inoculation (*P* = 0.003) after which population numbers declined. We then examined the rate of population decline between five days post inoculation and 100 days and found that the slopes were significantly different from zero (P<0.0001 in both cases; assumptions of normality and constant variance were met). Furthermore, the rate of decline in the H4H population was less severe when CFM was present compared to when CFM was absent (*P*<0.0006; Fig. 3) Based on the regression models, the average time required for the *E. coli* H4H population to drop below 10 cfu (based on the lower 95% confidence interval) is 106 days for 0 ppm CFM and 175 days for 50 ppm CFM treatments. Thus, presence of CFM in this experiment produced a significant numeric advantage and allowed the bacteria to persist at least two months longer than would occur in the absence of CFM.

### Cef^S^ *E. coli* Populations Decline in the Presence of Ceftiofur Metabolites

**Figure 4:**
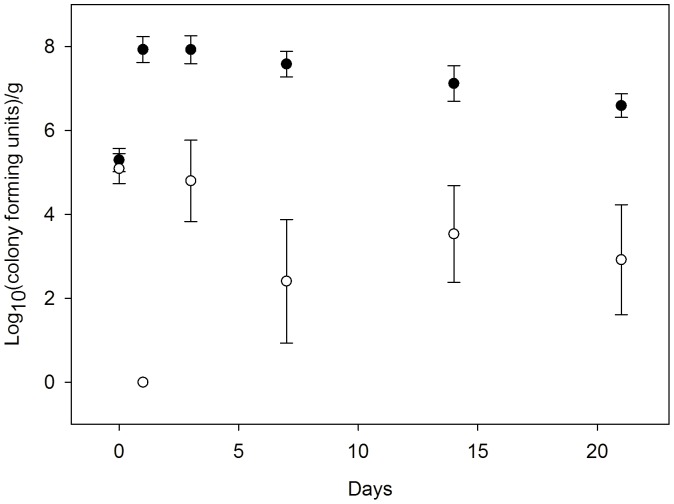
Ceftiofur metabolites confer selective advantage to cef^R^ *E. coli* by restricting cef^S^ population in soil. Mean colony forming units for presumptively ceftiofur sensitive (cef^S^) fecal *E. coli* in a matrix of sandy soil and feces (25∶1) after addition of urine containing 0 ppm (open circles) or 50 ppm (closed circles) CFM and incubated at 23°C. Bars = s.e.m.

To evaluate the impact of CFM on susceptible bacteria, we quantified changes in cfu for fecal *E. coli* in a soil and feces matrix with urine (0 or 50 ppm CFM) at 23°C (Fig. 4). No additional bacteria were added to the matrix. When CFM was included, no fecal *E. coli* were detected after 24 h. Nevertheless, cef^R^ *E. coli* were recovered from several of the replicate treatments at day 3 despite no detection of cef^R^ *E. coli* in the original matrices. The appearance of cef^R^ *E. coli* represents either enrichment of undetected subpopulations of cef^R^ *E. coli* or spontaneous resistance due to mutation of the *E. coli ampC* [22]. This experiment demonstrated that CFM can severely restrict CFM sensitive bacteria (Fig. 4) while allowing numerical enrichment of cef^R^ *E. coli* populations (Fig. 3).

### Cattle can Acquire Bacteria from Soil

To verify that environmental cef^R^ *E. coli* can colonize animals, we sprayed a suspension of cef^R^ *E. coli* onto compost bedding, allowed it to dry 30 min (1.6×10^9^ cfu/g), and then introduced dairy calves (n = 2). Calves received normal rations, feces were sampled directly from the rectum, and no antibiotics were administered. By day 2 both calves were shedding ∼10^5^ cfu/g. By day 7 calves were shedding ∼10^6^ cfu/g while the bedding count had dropped to ∼10^4^ cfu/g (Table 1). Strain identity was confirmed by antibiotic resistant pattern (cef^R^ and resistance to florfenicol, flo^R^). No cef^R^ flo^R^ *E. coli* were detected in a control room where unsprayed bedding was used (n = 2 calves).

**Table 1:** Calves acquire cef^R^ *E. coli* from bedding.

Days	Bedding cfu/g	Feces cfu/g
0	9.6	None
2	5.9	5±0.5
4	4.6	5.4±0.4
7	4.1	6.2±0.2

The log_10_ cfu (colony forming units) of *E. coli* strain AR060302 per g of bedding material and feces (n = 2 calves; ± s.e.m). The AR060302 *E. coli* population declined >5 log_10_ cfu/g in bedding material while increasing from below detectable level to >6 log_10_ cfu/g feces (mean ± s.e.m). No antibiotics were administered during the course of this experiment.

## Discussion

We show that CFM in urine remains bactericidal in soils mixed with feces for significantly longer duration (D_50_) at colder temperatures and at higher CFM concentrations. Biodegradation, adsorption to surfaces, abiotic hydrolysis and photodegradation are responsible for degradation of beta-lactam drugs (including ceftiofur) in feces, soil and water [19], [20], [23]. Our results implicate biodegradation as the major mechanism of CFM inactivation in soils. This was highlighted by the dramatic increase in D_50_ at 23°C when soil alone was autoclaved (Fig. 2); clearly heat-labile biological components of soil play a dominant role in the degradation of CFM. The extended persistence of bactericidal CFM at 4°C is also consistent with decreased biodegradation with reduced microbial metabolic activity, and *vice versa* at warmer temperatures. The reduced D_50_ in soil at 4°C with the addition of either fresh or autoclaved feces suggests increased soil microflora activity due to a nutrient effect [23], [24], [25] although fecal microflora may contribute to this process. Experimental chambers were devoid of light thereby excluding photodegradation in these experiments. Because CFM remains bactericidal in urine at both 23°C and 4°C for >110 days, the role of abiotic hydrolysis of CFM was minimal at best. It is notable, however, that higher temperature and pH will enhance abiotic hydrolysis of ceftiofur [19], [20].

Reduced biodegradation at lower temperatures means CFM loading will be greater during colder seasons. If true, there may be a significantly greater opportunity to enrich cef^R^ bacterial populations with the onset of warmer temperatures when the load of CFM is potentially highest. This will be influenced, of course, by the frequency of ceftiofur administration during the course of the year and other factors, such as substrate removal during pen cleaning. Importantly, even a brief exposure (∼2.7±0.3 days) to CFM is sufficient to confer a numerical advantage (>1 log_10_) and extended persistence for cef^R^ *E. coli* in soil. In our model persistence was extended approximately two months at 23°C, well after the ∼3 days when CFM could exert a direct biological impact. This advantage likely arises because of selection against the co-resident competitors (Fig. 4) [26], [27], [28]. Hammesfahr et al. [26] found that addition of pig manure contaminated with sulfadiazine to soil resulted in a reduction of soil bacteria/fungi ratio. In addition, autoclaving cattle manure confers numerical and extended survivorship advantages to *Salmonella* sp. and *E. coli* O157:H7 by reducing the competitive bacterial populations [27], [28]. The numerical enrichment of H4H *E. coli* and fecal coliform populations on the first day of our experiments also indicates the possible role of urine as a nutrient source in the matrix. This is consistent with the previous findings where the addition of urine in wood chips favored the growth of *E*. *coli* O157:H7 [29].

Even if CFM enriches cef^R^ populations in soil this would only be important if these resistant bacteria could be transmitted back to livestock. Our bedding exposure study clearly illustrates that transmission is feasible, and others have also reported transmission of bacteria through simple contact with the floor, hide, food and water [29], [30], [31]. Consequently, the dramatic expansion of cef^R^ enteric bacteria in food animal populations could be explained in part through a process of environmental selection and transmission back to food animals; the effect shown here is much more dramatic than has been reported for *in vivo* selection following parenteral administration of ceftiofur [8], [10], [13], [14], [17]. While we designed these experiments to reflect physiologically relevant metabolites and concentrations, our bioassay only measured the biological effect of CFM, but not the actual concentration of CFM. Ideally, measurements under field conditions would include both analytic and biological assays.

Despite the clear treatment effects demonstrated in this study, the artificial nature of these experiments needs to be emphasized. Under natural field conditions the heterogeneity of drug distribution and a multitude of physical, chemical, and biological factors could enhance or diminish the rates and magnitudes of the effects described herein. Clearly, more study is needed. Nevertheless, if our findings are generalizable to field conditions it is useful to consider the environment as another point of intervention to limit the proliferation of antibiotic resistance bacteria. For example, our findings highlight the possibility that engineered solutions could be developed so that this important veterinary drug can be used without perpetuating resistance in non-target enteric bacteria. This might involve bioremediation, addition of adsorption agents, or improved waste management. Such strategies might also mitigate selection from other excreted antibiotics that remain bioavailable in the environment.

## References

[1] FDA (2012) New Animal Drugs; Cephalosporin Drugs; Extralabel Animal Drug Use; Order of Prohibition. Federal Register 77: 735–745.

[2] Call DR, Singer RS, Meng D, Broschat SL, Orfe LH, et al (2010) *Bla* _CMY-2_-positive IncA/C plasmids from *Escherichia coli* and *Salmonella enterica* are a distinct component of a larger lineage of plasmids. Antimicrob Agents Chemother 54: 590–596. 19949054 10.1128/AAC.00055-09 PMC2812137

[3] Giles WP, Benson AK, Olson ME, Hutkins RW, Whichard JM, et al (2004) DNA sequence analysis of regions surrounding *bla* _CMY-2_ from multiple *Salmonella* plasmid backbones. Antimicrob Agents Chemother 48: 2845–2852. 15273090 10.1128/AAC.48.8.2845-2852.2004 PMC478531

[4] Winokur PL, Brueggemann A, DeSalvo DL, Hoffmann L, Apley MD, et al (2000) Animal and human multidrug-resistant, cephalosporin-resistant *Salmonella* isolates expressing a plasmid-mediated CMY-2 AmpC beta-lactamase. Antimicrob Agents Chemother 44: 2777–2783. 10991860 10.1128/aac.44.10.2777-2783.2000 PMC90151

[5] Donaldson SC, Straley BA, Hegde NV, Sawant AA, DebRoy C, et al (2006) Molecular epidemiology of ceftiofur-resistant *Escherichia coli* isolates from dairy calves. Appl Environ Microbiol 72: 3940–3948. 16751500 10.1128/AEM.02770-05 PMC1489609

[6] Lindsey RL, Fedorka-Cray PJ, Frye JG, Meinersmann RJ (2009) Inc A/C plasmids are prevalent in multidrug-resistant *Salmonella enterica* isolates. Appl Environ Microbiol 75: 1908–1915. 19181840 10.1128/AEM.02228-08 PMC2663206

[7] Sawant AA, Hegde NV, Straley BA, Donaldson SC, Love BC, et al (2007) Antimicrobial-resistant enteric bacteria from dairy cattle. Appl Environ Microbiol 73: 156–163. 17098918 10.1128/AEM.01551-06 PMC1797124

[8] Heider LC, Funk JA, Hoet AE, Meiring RW, Gebreyes WA, et al (2009) Identification of *Escherichia coli* and *Salmonella enterica* organisms with reduced susceptibility to ceftriaxone from fecal samples of cows in dairy herds. Am J Vet Res 70: 389–393. 19254152 10.2460/ajvr.70.3.389

[9] Tragesser LA, Wittum TE, Funk JA, Winokur PL, Rajala-Schultz PJ (2006) Association between ceftiofur use and isolation of *Escherichia coli* with reduced susceptibility to ceftriaxone from fecal samples of dairy cows. Am J Vet Res 67: 1696–1700. 17014318 10.2460/ajvr.67.10.1696

[10] Daniels JB, Call DR, Hancock D, Sischo WM, Baker K, et al (2009) Role of ceftiofur in selection and dissemination of *bla* _CMY-2_-mediated cephalosporin resistance in *Salmonella enterica* and commensal *Escherichia coli* isolates from cattle. Appl Environ Microbiol 75: 3648–3655. 19376926 10.1128/AEM.02435-08 PMC2687309

[11] USDA (2007) Bacterial Epidemiology and Antimicrobial Resistance Research Unit, United States Department of Agriculture Russell Research Center, Athens, Georgia. 2007 Veterinary Isolates Final Report, Table 4. Available: http://www.ars.usda.gov/Main/docs.htm?docid=17649&page=5. Accessed 2012 Oct 11.

[12] Subbiah M, Top EM, Shah DH, Call DR (2011a) Selection pressure required for long-term persistence of *bla* _CMY-2_-positive IncA/C plasmids. Appl Environ Microbiol 77: 4486–4493. 21602382 10.1128/AEM.02788-10 PMC3127679

[13] Jiang X, Yang H, Dettman B, Doyle MP (2006) Analysis of fecal microbial flora for antibiotic resistance in ceftiofur-treated calves. Foodborne Pathog Dis 3: 355–365. 17199517 10.1089/fpd.2006.3.355

[14] Lowrance TC, Loneragan GH, Kunze DJ, Platt TM, Ives SE, et al (2007) Changes in antimicrobial susceptibility in a population of *Escherichia coli* isolated from feedlot cattle administered ceftiofur crystalline-free acid. Am J Vet Res 68: 501–507. 17472449 10.2460/ajvr.68.5.501

[15] Hornish RE, Kotarski SF (2002) Cephalosporins in veterinary medicine - ceftiofur use in food animals. Curr Top Med Chem 2: 717–731. 12052187 10.2174/1568026023393679

[16] Davis MA, Hancock DD, Besser TE, Daniels JB, Baker KN, et al (2007) Antimicrobial resistance in *Salmonella enterica* serovar Dublin isolates from beef and dairy sources. Vet Microbiol 119: 221–230. 17034963 10.1016/j.vetmic.2006.08.028

[17] Singer RS, Patterson SK, Wallace RL (2008) Effects of therapeutic ceftiofur administration to dairy cattle on *Escherichia coli* dynamics in the intestinal tract. Appl Environ Microbiol 74: 6956–6962. 18820057 10.1128/AEM.01241-08 PMC2583494

[18] Jaglan PS, Kubicek MF, Arnold TS, Cox BL, Robins RH, et al (1989) Metabolism of ceftiofur. Nature of urinary and plasma metabolites in rats and cattle. J Agri Food Chem 37: 1112–1118.

[19] Gilbertson TJ, Hornish RE, Jaglan PS, Koshy KT, Nappier JL, et al (1990) Environmental fate of ceftiofur sodium, a cephalosporin antibiotics. Role of animal excreta in its decomposition. J Agri Food Chem 38: 890–894.

[20] Subbiah M, Mitchell SM, Ullman JL, Call DR (2011b) β-lactams and florfenicol antibiotics remain bioactive in soils while ciprofloxacin, neomycin, and tetracycline are neutralized. Appl Environ Microbiol 77: 7255–7260. 21856822 10.1128/AEM.05352-11 PMC3194842

[21] El-Gendy AAM, Tohamy MA, Ismail M (2007) Comparative pharmacokinetic and renal clearance study of ceftiofur in cross breed Friesian and buffalo calves. BS VetMedJ 17: 69–77.

[22] Caroff N, Espaze E, Gautreau D, Richet H, Reynaud A (2000) Analysis of the effects of-42 and-32 *ampC* promoter mutations in clinical isolates of *Escherichia coli* hyperproducing AmpC. Journal of Antimicrobial Chemotherapy 45: 783–788. 10837430 10.1093/jac/45.6.783

[23] Jiang M, Wang L, Ji R (2010) Biotic and abiotic degradation of four cephalosporin antibiotics in a lake surface water and sediment. Chemosphere 80: 1399–1405. 20579689 10.1016/j.chemosphere.2010.05.048

[24] Schmitt H, Haapakanga H, Beelen PV (2005) Effects of antibiotics on soil microorganisms: time and nutrients influence pollution-induced community tolerance. Soil Biol Biochem 37: 1882–1892.

[25] Rafii F, Williams AJ, Park M, Sims LM, Heinze TM, et al (2009) Isolation of bacterial strains from bovine fecal microflora capable of degradation of ceftiofur. Vet Microbiol 139: 89–96. 19428193 10.1016/j.vetmic.2009.04.023

[26] Hammesfahr U, Kotzerke A, Lamshoft M, Wilke BM, Kandeler E, et al (2011) Effects of sulfadiazine-contaminated fresh and stored manure on a soil microbial community. Eur J Soil Biol 47: 61–68.

[27] Jiang X, Morgan J, Doyle MP (2002) Fate of *Escherichia coli* O157:H7 in manure-amended soil. Appl Environ Microbiol 68: 2605–2609. 11976144 10.1128/AEM.68.5.2605-2609.2002 PMC127522

[28] You Y, Rankin SC, Aceto HW, Benson CE, Toth JD, et al (2006) Survival of *Salmonella enterica* serovar Newport in manure and manure-amended soils. Appl Environ Microbiol 72: 5777–5783. 16957193 10.1128/AEM.00791-06 PMC1563654

[29] Davis MA, Cloud-Hansen KA, Carpenter J, Hovde CJ (2005) *Escherichia coli* O157:H7 in environments of culture-positive cattle. Appl Environ Microbiol 71: 6816–6822. 16269714 10.1128/AEM.71.11.6816-6822.2005 PMC1287631

[30] Rasmussen MA, Casey TA (2001) Environmental and food safety aspects of *Escherichia coli* O157:H7 infections in cattle. Crit Rev Microbiol 27: 57–73. 11450854 10.1080/20014091096701

[31] Stanford K, Stephens TP, McAllister TA (2010) Use of model super-shedders to define the role of pen floor and hide contamination in the transmission of *Escherichia coli* O157:H7. J Anim Sci 89: 237–244. 20852081 10.2527/jas.2010-3088

